# Systematic Review and Meta-Analysis of Randomized Controlled Trials of Xingnaojing Treatment for Stroke

**DOI:** 10.1155/2014/210851

**Published:** 2014-02-24

**Authors:** Weijun Peng, Jingjing Yang, Yang Wang, Weihao Wang, Jianxia Xu, Lexing Wang, Zhihua Xing

**Affiliations:** ^1^Institute of Integrated Medicine, Xiangya Hospital, Central South University, No. 87 Xiangya Road, Changsha, Hunan 410008, China; ^2^Institute of Integrated Medicine, Hunan Provincial Tumor Hospital, Changsha 410013, China; ^3^Institute of Integrated Medicine, The Affiliated Tumor Hospital of Xiangya Medical School, Central South University, Changsha 410013, China

## Abstract

*Objective*. Xingnaojing injection (XNJ) is a well-known traditional Chinese patent medicine (TCPM) for stroke. The aim of this study is to assess the efficacy of XNJ for stroke including ischemic stroke, intracerebral hemorrhage (ICH), and subarachnoid hemorrhage (SAH). *Methods*. An extensive search was performed within using eight databases up to November 2013. Randomized controlled trials (RCTs) on XNJ for treatment of stroke were collected. Study selection, data extraction, quality assessment, and meta-analysis were conducted according to the Cochrane standards, and RevMan5.0 was used for meta-analysis. *Results*. This review included 13 RCTs and a total of 1,514 subjects. The overall methodological quality was poor. The meta-analysis showed that XNJ combined with conventional treatment was more effective for total efficacy, neurological deficit improvement, and reduction of TNF-**α** levels compared with those of conventional treatment alone. Three trials reported adverse events, of these one trial reported mild impairment of kidney and liver function, whereas the other two studies failed to report specific adverse events. *Conclusion*. Despite the limitations of this review, we suggest that XNJ in combination with conventional medicines might be beneficial for the treatment of stroke. Currently there are various methodological problems in the studies. Therefore, high-quality, large-scale RCTs are urgently needed.

## 1. Introduction

Stroke is the second most common cause of death worldwide and a major global cause of disability. Its increasing global impact in the future decades ahead is predicted to be the greatest in middle-income countries, including China. Approximately one-fifth of the world's total population resides in China, where stroke is already the leading cause of death and adult disability [1–3]. The costs of care, lost productivity, and premature mortality are high for stroke survivors; the direct and indirect costs in the United States were $38.6 billion in 2008 [4]. It is becoming increasingly recognized that modern Western medicine may sometimes fall short of desired goals in its standardized treatments for specific illnesses, whereas complementary medicine based on different theories can offer substantial improvements for such illnesses [5].

Traditional Chinese medicine (TCM), including herbal medicines and other nonmedication therapies, has been used to treat stroke in China during the past 2,000 years [5–9]. In recent decades, advances in the pharmaceutical industry have led to the development of traditional Chinese patent medicine (TCPM), which has been routinely used for treating stroke patients in hospitals practicing either Western medicine or traditional Chinese medicine [6, 10, 11]. Xingnaojing injection (XNJ), which is composed of four medicinal herbs including artificial musk, synthetic borneol, *Curcuma aromatica* Salisb, and *Gardenia jasminoides* J.Ellis, is an effective TCPM, that is extracted using modern biotechnology according to TCM named *An Gong Niu Huang Wan* [12]. Studies have reported that XNJ can reduce brain injury and enhance functional recovery after stroke in different clinical trials and animal models of injury [13–16]. Several previous systematic reviews published in Chinese academic journals have reported the effects of XNJ for ischemic stroke or ICH [17–23]. However, no published systematic reviews have summarized the existing evidences including the three primary pathologic types of stroke (ischemic stroke, ICH, and SAH) [24], and no systematic reviews of the effectiveness and safety of XNJ treatment for stroke have been published in English academic journals. Furthermore, various new related trials have been published. New clinical trials have been published recently, and these data have not been included in a systematic meta-analysis of XNJ treatment for stroke.

The aim of our study was to perform a comprehensive systematic review and meta-analysis of all the published randomized controlled trials (RCTs) to compare the efficacy of XNJ plus conventional medicine compared with that of conventional medicine alone in the treatment of stroke.

## 2. Methods

### 2.1. The Literature Search

All RCTs published on the efficacy of XNJ in treating stroke were retrieved from eight bibliographical databases including Medline (1950 to November 2013), PubMed (1982 to 2013), the Cochrane Library (Issue 3, 2013), ScienceDirect (1960 to 2013), Embase (1966 to November 2013), China National Knowledge Infrastructure (CNKI; 1994 to November 2013), Wan-Fang Data (1989 to November 2013), and Vip (1989 to November 2013). All database entries were scanned by keyword search, from the database inception dates up to the last search that was performed on 30 November 2013. A simple combinatorial keyword search strategy was used, which searched for the keyword “Xingnaojing” combined with one of the keywords “stroke,” “ischemic stroke,” “ICH,” or “SAH.” Full details on the search terms are described in the appendix Exact search strategies (terms) and query syntax for specific databases were adapted according to the same keyword strategy.

### 2.2. Study Selection

The six criteria used for inclusion of each identified RCT study were as follows: (1) participants had to be within 48 hours of stroke onset, and stroke diagnosis had to be confirmed with computed tomography/magnetic resonance imaging (CT/MRI); (2) studies had to be definite randomized controlled trials (trials with a clear method of randomization) for evaluating the effects of XNJ on stroke; (3) studies had to investigate the efficacy of XNJ alone or in combination with conventional Western medicine and compared with results using conventional Western or traditional Chinese medicines (that did not include XNJ); (4) treatments had to last for 14 days or more; (5) more than 60 participants had to be included in the study; and (6) studies had to include one of the defined outcome measures (primary outcome measures were the total effective rate, and secondary outcome measures were neurological deficit scores (based on NIHSS, ESSS, and CSS standards) and inflammation factors).

The three criteria used for exclusion of each identified RCT study were as follows: (1) the study was a duplicated or redundant publication; (2) the study was based only on animal models; and (3) the diagnosed acute cerebrovascular disease, cerebral hemorrhage, or cerebral infarction was induced by traumatic brain injury, intracranial vascular malformation, intracranial aneurysm, extracranial dynamic artery (vein) disease, or cerebral arteritis.

**Figure 1:**
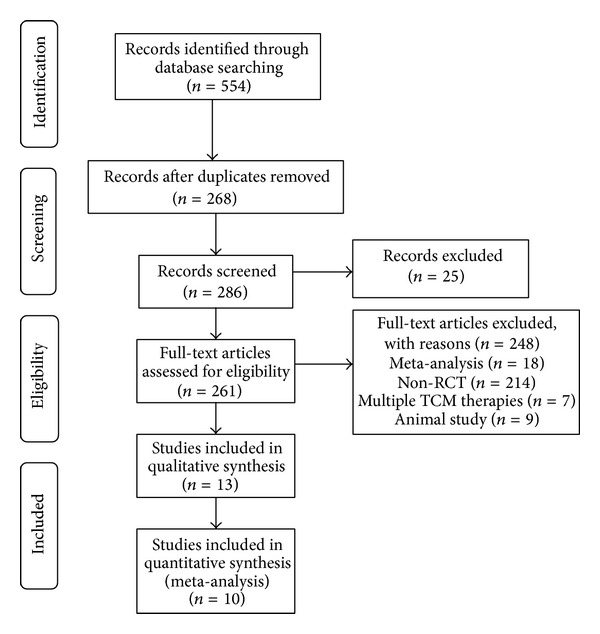
Flowchart of the literature search and study selection.

Two investigators (W. J. Peng and Y. Wang) independently searched the databases and selected studies according to the inclusion and exclusion criteria. Disagreements between the reviewers were resolved by consensus after discussion.  Figure 1 shows a flow diagram of the study selection.

### 2.3. Data Extraction

Two investigators (J. J. Yang and Y. Wang) independently extracted the following data from the selected studies: (1) year of publication, (2) follow-up periods, (3) baseline characteristics for participants in different groups, (4) cohort sizes, (5) outcome measures, (6) dosages, (7) types of stroke, and (8) adverse events.

### 2.4. Quality Assessment of Selected Studies

The quality assessment of the trials selected for inclusion was evaluated using the Jadad score [25]. The Jadad score consisted of three items: randomization (0–2 points), blinding (0–2 points), and dropouts and withdrawals (0-1 points). The response to each item was either “yes” (1 point) or “no” (0 points). The final score ranged from 0 to 5 points, with higher scores indicating better reporting. Studies with a Jadad score of 2 or less were considered to have low quality and those with a Jadad score of 3 or more were considered to have high quality [26].

The Cochrane Collaboration's tool was used to assess the risk of bias in the studies. Risk of bias was assessed independently and in duplicate by two investigators (W. J. Peng and J. X. Xu) using the following seven criteria: (1) random sequence generation, (2) allocation concealment (selection bias), (3) blinding of participants and personnel (performance bias), (4) blinding of outcome assessment (detection bias), (5) incomplete outcome data (attrition bias), (6) selective reporting (reporting bias), and (7) other bias [27]. The response for each criterion was reported as low risk of bias, high risk of bias, and unclear risk of bias. Any disagreement between the investigators was resolved with mutual consensus in the presence of a third investigator. To verify unclear information on methodology and therapy, attempts were made to contact the authors of the original papers via phone or e-mail. If the authors could not be contacted after three attempts, the studies were excluded.

### 2.5. Statistical Analysis

Data were processed in accordance with the Cochrane Handbook. RevMan version 5.0 (Cochrane Collaboration, Copenhagen, Denmark) was used to combine results from ≥2 separate trials. Before the results of the studies were combined, statistical heterogeneity among studies was estimated using the chi-square test and *I* ^2^ test (*P* > 0.05 and *I* ^2^ < 50% indicate acceptable heterogeneity between the pooled studies) [28]. The fixed-effects model was used first for meta-analysis; if heterogeneity was present, the random-effects model would have been used. Intervention effects were expressed with odds ratio (OR), and the associated 95% confidence intervals (CIs) were calculated for dichotomous outcomes. The standard mean difference (SMD) or weighted mean difference (WMD) was calculated for continuous outcomes that were measured using the same methodology.

## 3. Results

### 3.1. Study Selection

We identified and selected 554 papers by performing keyword searches of titles and abstracts, including 208 records from CNKI, 166 from Wan-Fang Data, 174 from Vip, 1 from ScienceDirect, 3 from the Cochrane Library, and 2 from PubMed. According to the specified selection criteria (described in Methods, Section 2.2), 13 studies [13, 29–40] were included for further quality assessment. This yielded a total of 10 [13, 29–31, 33–35, 37, 38, 40] that were utilized for meta-analysis. The screening process is summarized in a flowchart in Figure 1.

### 3.2. Study Characteristics

Table 1 lists the main characteristics of the final 13 studies that were selected for inclusion. These studies were published in Chinese academic journals between 2000 and 2013, including a total of 1,714 participants, and each study was performed at a single center. The follow-up periods ranged from 14 to 28 days. XNJ was compared with conventional treatments in these RCTs. The drugs administered to the control groups included primarily citicoline, piracetam, mannitol, statins, platelet aggregation blockers, and some conventional Chinese medicinal products for treating stroke (e.g., Danshen injection). All 13 RCTs administered XNJ plus the conventional treatments to the test groups, whereas the conventional treatments alone were administered to the control groups. For outcome measures, clinical effective rate was measured in 6 studies [13, 29, 30, 33, 37, 40], neurological deficit score was measured in 7 studies [13, 31, 32, 34–36, 38], and the level of TNF-*α* was measured in 3 studies [31, 34, 35].

### 3.3. Quality Assessment of Selected Studies

The quality assessment of the trials selected for inclusion was performed using the Jadad score and the Cochrane Collaboration's tool. The detailed results are presented in Table 2. Using the Jadad scale, two studies [29, 31] obtained a score of 3, whereas the remaining studies obtained a score of 2. The Cochrane Collaboration's tool for quality assessment revealed the following bias risks in the studies: (1) there was a low risk for selection bias in random sequence generation, and incomplete outcome data corrected for attrition bias; (2) there were high risks for selection bias in allocation concealment and blinding of participants, performance bias for personnel, and detection bias for blinding outcome assessment in patient-reported outcomes; (3) there was an unclear risk for reporting bias in the self-selected sources of bias that were reported in each study.

### 3.4. Meta-Analysis

#### 3.4.1. Total Effective Rate of Xingnaojing

**Figure 2:**
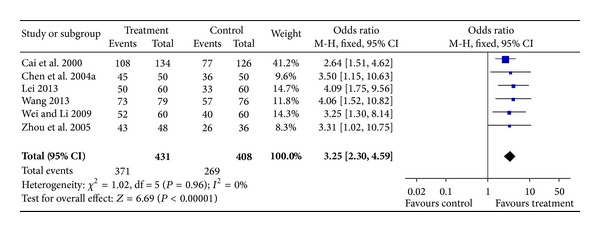
Forest plot of comparison: the total effective rate.

Six trials [13, 29, 30, 33, 37, 40] involving 839 patients calculated the total effective rate of Xingnaojing, which evaluated the measured outcome on the basis of the neurological deficit score and the general neurological status in acute stroke patients [10, 41]. The fixed-effects model was used for statistical analysis. The pooled analysis indicated that XNJ in combination with conventional medicine improved the clinical efficacy rates and patient outcomes when compared with those for patients in the control groups (OR = 3.25; 95% CI, 2.30–4.59; *P* < 0.00001). There was no evidence for heterogeneity between comparisons (chi-square = 1.02; degrees of freedom (df) = 5 (*P* = 0.96); *I* ^2^ = 0%) (Figure 2).

#### 3.4.2. The Neurological Deficit Score

**Figure 3:**
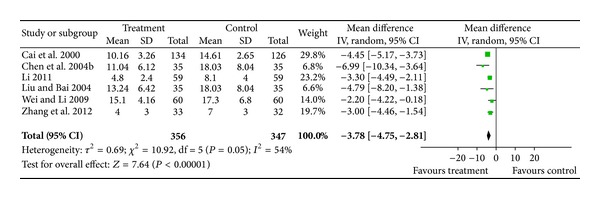
Forest plot of comparison: the neurological deficit score.

Six included trials [13, 29, 31, 34, 35, 38] involving 703 patients measured functional outcomes for 14 days after stroke. Two additional studies [32, 36] that measured poststroke functional outcomes were excluded due to different assessment time points (7 or 21 days), and another study [39] was excluded due to duplication. The random-effects model was used for statistical analysis. The pooled analysis indicated that patients in the treatment groups had significantly lower neurological deficit scores than patients in the control groups (WMD = −3.78; 95% CI, −4.75 to −2.81; *P* < 0.00001). There was evidence for statistical heterogeneity between comparisons (chi-square = 10.92; df = 5 (*P* = 0.05); *I* ^2^ = 54%) (Figure 3).

#### 3.4.3. Levels of TNF-*α* in Serum

**Figure 4:**
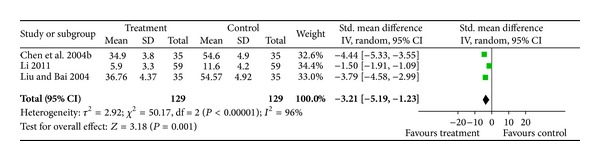
Forest plot of comparison: TNF-*α*.

Three studies [31, 34, 35] involving 258 patients measured the levels of TNF-*α* in serum for 14 days after stroke. The random-effects model was used for statistical analysis. The pooled analysis indicated that there was a significant difference in the serum levels of TNF-*α* between the treatment and control groups (SMD = −3.21; 95% CI, −5.19 to −1.23; *P* = 0.001). There was evidence for statistical heterogeneity between comparisons (chi-square = 50.17; df = 2 (*P* < 0.00001); *I* ^2^ = 96%) (Figure 4).

### 3.5. Possible Drug Protection Mechanism Analysis

Ten [29–36, 38, 39] of the 13 studies selected during initial screening assessed the biological mechanisms of XNJ activity. A wide variety of possible neuroprotective mechanisms were proposed within these studies. The neuroprotective effect of XNJ was attributed primarily to improvement of cerebral circulation and blood flow and a reduction of cerebral edema, ferritin, and inflammation. In addition, XNJ may promote angiogenesis. The potential association of XNJ with anti-inflammatory effects received the greatest attention in the studies (Table 3).

### 3.6. Adverse Events

Three trials [13, 32, 33] reported adverse events, Chen [32] reported that the most common adverse effect of XNJ treatment was mild impairment of kidney and liver function; however, the impairment resolved without special treatment. The other two studies failed to report specific adverse events. No life-threatening adverse effects were reported in these studies.

### 3.7. Publication Bias

Publication bias could not be assessed because of the small number of studies (<10 studies) [42, 43]. We carefully selected the trials and excluded studies with measurable bias. However, all of the assessed trials were published in Chinese academic journals. Therefore, the potential for publication bias cannot be excluded.

## 4. Discussion

Improved pharmaceutical technologies in China have led to the development of many TCPM oral agents and injections for the prevention and treatment of stroke that are based on famous traditional Chinese medical prescriptions. Currently, there are more than 60 TCPM treatments for stroke that have been approved by the Chinese State Food and Drug Administration for clinical practice during the past 30 years. However, few studies have evaluated their effectiveness according to current rigorous international standards [6, 10, 11]. Most of the systematic reviews published in Chinese academic journals report the efficacy of the XNJ for ischemic stroke or ICH but not for the combination of ischemic stroke with ICH or SAH. This review was intended to provide an internationally accessible systematic review of the clinical efficacy and safety of XNJ for the three main pathological types of stroke.

The present investigation analyzed data from 13 RCTs involving 1,514 individuals that investigated the use of XNJ for the treatment of stroke. We evaluated the total effective rate of XNJ and found that XNJ plus conventional treatment was more effective for stroke than conventional treatment alone. Two trials [13, 23] reported efficacy for patients revived from a stroke-induced coma, and the results showed that more patients with coma regained consciousness within two weeks in the XNJ groups compared to that in the control groups. We found that XNJ may have greater efficacy for improving neurological deficit and reducing the serum level of TNF-*α* compared to those in control groups. These results were encouraging and showed the potential benefit of combining XNJ with conventional medicines for the treatment of stroke. These results are consistent with the effects reported for XNJ in previous animal studies and justify further clinical trials.

Adverse events were reported by three trials [13, 32, 33], but no life-threatening adverse effects were observed. However, conclusions regarding the safety of XNJ cannot be determined from our meta-analysis due to the limited evidence provided by the eligible trials. The use of XNJ for treating acute ICH may raise a concern because the two XNJ components: artificial musk and *Curcuma aromatica* Salisb promote blood circulation and remove blood stasis. To properly assess the safety of XNJ, large-scale clinical trials with long-term follow-up are required.

The overall methodological quality of the RCTs was judged to be poor. The trials selected for inclusion contained some methodological deficiencies that could lead to a high risk of bias. Many studies failed to describe their methods in detail. For example, although all trials claimed randomization, most trials failed to provide enough information to judge whether the randomization procedures had been carried out properly. Only two studies [29, 32] claimed single blinding, and no studies reported double blinding. The strategy of double blinding is a necessary element in a clinical trial that prevents the appearance of the placebo effect, which tends to favor the treatment and produces a false-positive conclusion. We also found that the reporting of trial procedures was frequently unclear and insufficient. Consequently, the results should be interpreted with caution, which limits the value of conclusions about the overall efficacy of XNJ for treating stroke. The potential benefits of XNJ for treating stroke need to be further evaluated through clinical trials that employ rigorous methodologies. Therefore, we suggest that all reports of RCTs published in China should be required to comply with the CONSORT statement [44], and the publication of detailed protocols should be encouraged. These strategies would lead to the identification and use of many TCPM treatments for stroke outside China.

Surprisingly, we found an abnormal incidence of duplication when we evaluated the initially selected papers. Two studies were considered to have strong probability of plagiarism, which was revealed by comparing the texts. Therefore, it is important for authors or reviewers of systematic meta-analyses to identify and remove duplicated and plagiarized studies, to avoid inflating the number of studies and misrepresenting the apparent efficacy of drugs, and to reduce the heterogeneity of the analyses.

The current systematic reviews have various limitations. First, meta-analyses can only utilize available data, which are usually reported in published studies. Negative or neutral studies are less likely to be published, so the results from published data may overstate treatment efficacy. Second, inadequate reporting of allocation concealment, blinding, intention-to-treat, and dropouts in all the trials may have created potential performance and detection bias, because patients and researchers might have been aware of the therapeutic interventions. Third, all of the included studies were published in Chinese journals; all the results were positive, and the number of studies was not enough to implement a funnel plot, so there might be an undetected publication bias. We could not rule out systematic error, because the cohort sizes of all studies were limited. Additionally, heterogeneity must be considered for any meta-analysis. The main reasons for heterogeneity were the limited number of trials and small cohorts; therefore, additional large-scale clinical trials are required. Another important reason for the existence of heterogeneity was due to the low quality and potential bias of the trials selected for analysis. That the heterogeneity is surprisingly low in meta-analysis of the total effective rate required to be considered. It would also be associated with the poor methodological quality of the selected trials, which need to be further investigated. Finally, none of the selected trials reported quality-of-life or cost-effectiveness considerations, which are clear benefits conferred by TCM.

Future randomized controlled trials of TCPM should incorporate improved methodological reporting and quality control as follows: (1) all clinical trials should be registered and comply with guidelines in the CONSORT statement [44]; (2) the cohort sizes required for statistical significance should be calculated; (3) adequate generation of the allocation sequence and adequate allocation concealment should be reported; (4) the blinding strategy should be reported clearly and in detail; (5) placebo control should be included; (6) the balance of basic demographic data and baseline disease should be stated; (7) standard, validated, and important clinical outcome measures such as death, dependency, and quality of life for at least 6-month follow-up should be reported, and the outcome assessment tools should be scientifically sounded in terms of reliability, validity, and responsiveness; (8) XNJ-related adverse events should be rigorously assessed by standardized monitoring and an effective self-reporting system.

In summary, a definite conclusion on the efficacy and adverse events associated with XNJ treatment of stroke cannot be drawn from this systematic review because of the poor methodological quality of the RCT trials. We cautiously suggest that XNJ combined with conventional treatment was more effective for the treatment of stroke with respect to the primary outcome (the total effective rate) and the secondary outcomes (neurological deficit and inflammation factors) compared with those in the control groups. Future systematic analyses require additional RCTs with more rigorous experimental design, stronger quality control, longer follow-up periods, larger cohort sizes, and multicenter or international collaboration.

## Figures and Tables

**Table 1:** Characteristics of the included studies.

Study	Number of patients	Follow-up (days)	Baseline comparison	Adverse event	Outcomes measure (primary/secondary)	Treatment group dosage	Types of stroke
Cai et al. 2000 [29]	256	14	Y	N	TER, CSS	XNJ 30–60 mL/d + CWM	IS
Chen et al. 2004a [30]	100	14	Y	N	TER	XNJ 20 mL/d + CWM	IS
Chen et al. 2004b [31]	70	14	Y	N	TNF-*α*, IL-6, IL-1, ESSS	XNJ 40 mL/d + CWM	ICH
Chen 2013 [32]	202	21	Y	Y	NIHSS, IL-9	XNJ 30 mL/d + CWM	ICH
Lei 2013 [33]	120	28	Y	Y	TER	XNJ 20 mL/d + CWM	ICH
Li 2011 [34]	118	14	Y	N	TNF-*α*, IL-6, NIHSS	XNJ 40 mL/d + CWM	ICH
Liu and Bai 2004 [35]	70	14	Y	N	TNF-*α*, IL-6, IL-8, ESSS	XNJ 40 mL/d + CWM	ICH/IS
Ming et al. 2010 [36]	91	21	Y	N	ESS,	XNJ 20 mL/d + CWM	ICH
Wang 2013 [37]	155	14	Y	N	TER	XNJ 20 mL/d + CWM	ICH/IS/SAH
Wei and Li 2009 [13]	120	28	Y	Y	TER, CSS	XNJ 10 mL/d + CWM	ICH
Zhang et al. 2012 [38]	65	14	Y	N	NIHSS	XNJ 20 mL/d + CWM	IS
Zhang et al. 2013 [39]	63	14	Y	N	NIHSS	XNJ 20 mL/d + CWM	IS
Zhou et al. 2005 [40]	84	28	Y	N	TER	XNJ 20 mL/d + CWM	ICH

Note. XNJ is Xingnaojing injection; CWM is conventional western medicine; TER is total effective rate; SR is soberness rat; NIHSS is the National Institute of Health stroke scale; SSS is Scandinavian Stroke Scale; ESS is the European stroke scale; ESSS is Edinburgh-Scandinavia stroke scale; CSS is the Chinese stroke scale; IS is ischemic stroke; ICH is intracerebral hemorrhage; SAH is subarachnoid hemorrhage. The column of “Baseline comparison” shows that the study did (Y) or did not (N) report the baseline comparison between the treatment and control groups.

**Table 2:** Quality assessment of included studies.

Study	Random sequence generation	Allocation concealment	Blinding of participants and personnel	Blinding of outcome assessment	Incomplete outcome data	Selective reporting	Other bias	Jadad scores
Cai et al. 2000 [29]	L	H	L	H	L	U	L	3
Chen et al. 2004a [30]	L	H	H	H	L	U	U	2
Chen et al. 2004b [31]	L	H	H	H	L	U	H	3
Chen 2013 [32]	L	H	L	H	L	U	U	2
Lei 2013 [33]	L	H	H	H	L	U	H	2
Li 2011 [34]	L	H	H	H	L	U	H	2
Liu and Bai 2004 [35]	L	H	H	H	L	U	L	2
Ming et al. 2010 [36]	L	H	H	H	L	U	U	2
Wang 2013 [37]	L	H	H	H	L	U	U	2
Wei and Li 2009 [13]	L	H	H	H	L	U	H	2
Zhang et al. 2012 [38]	L	H	H	H	L	U	H	2
Zhang et al. 2013 [39]	L	H	H	H	L	U	U	2
Zhou et al. 2005 [40]	L	L	H	H	L	U	H	2

Note. L is low risk of bias; H is high risk of bias; U is unclear risk of bias.

**Table 3:** Possible protective mechanisms of XNJ.

Possible drug protective mechanisms	Studies
Cerebral edema relief	[34, 36]
Lesion volume reduction	[38, 39]
Endothelial function improvement	[30]
Anti-inflammatory effect	[31, 32, 34, 35]
Promotion of new blood vessel formation	[38, 39]
Reduction of ferritin	[33]
Hypothalamic-pituitary-adrenal cortex axis	[29]

**Table 4:** Search terms.

In English databases	In Chinese databases
Xingnaojing	Xingnaojing
Stroke	Zhong Feng (stroke)
Ischemic stroke	Chu Xue Xing Zhong Feng (ICH)
Intracerebral hemorrhage	Que Xue Xing Zhong Feng (ischemic stroke)
ICH	Nao Chu Xue (ICH)
Subarachnoid hemorrhage	Nao Geng Si (ischemic stroke)
SAH	Nao Geng Sai (ischemic stroke)
	Qiang Xi Xing Nao Geng Sai (ischemic stroke)
	Zhu Wang Mo Xia Qiang Chu Xue (SAH)

## Appendix

See Table 4.
